# Diagnostic Certainty in Characterizing Liver Lesions in Rectal Cancer: Abbreviated Liver MRI versus CT

**DOI:** 10.1245/s10434-024-16468-2

**Published:** 2025-01-21

**Authors:** Anita Wale, Heather Harris, Gina Brown

**Affiliations:** 1 Department of Radiology, St George’s Hospital NHS Foundation Trust, Cardiovascular and Genomics Research Institute, St George’s University of London, London, UK; 2 Department of Radiology, Chesterfield Royal Hospital NHS Foundation Trust, Chesterfield, UK; 3 Department of Surgery and Cancer, Imperial College London, London, UK

## Abstract

**Background:**

Early diagnosis of metastases is crucial but routine staging with contrast-enhanced multidetector computed tomography (ceMDCT) is suboptimal. A total of 20% will have indeterminate or too small to characterize (TSTC) liver lesions on CT, requiring formal characterization by magnetic resonance imaging (MRI). This UK cross-sectional study reports our experience undertaking routine abbreviated liver MRI (MRI).

**Patients and Methods:**

A total of 99 patients with rectal cancer had ceMDCT, abbreviated liver MRI, and rectal MRI at diagnosis. Liver imaging was scored for liver metastases, benign or indeterminate/TSTC lesions on a per patient basis. Primary rectal cancer was risk scored on MRI.

**Results:**

A total of 42/99 (42%) had liver lesion(s) on ceMDCT versus 55/99 (56%) by MRI, and 46/99 (46%) had high-risk rectal cancer. ceMDCT showed 5 patients with liver metastases, 14 with benign lesions, and 23 with indeterminate/TSTC lesions. MRI showed 6 with liver metastases, 45 with benign lesions, and 4 with indeterminate/TSTC lesions. All liver metastases were in high-risk rectal cancer, OR 17.18 (*p* = 0.06), with 12.5% conversion rate of TSTC lesions to metastases in high-risk rectal cancer and 0% in low-risk rectal cancer. Diagnostic certainty of the liver findings was achieved in 93% of patients by MRI compared with 45% by ceMDCT (*p* < 0.0001).

**Discussion:**

Abbreviated liver MRI diagnosed fewer indeterminate/TSTC lesions and provided greater diagnostic certainty than ceMDCT, *p* < 0.0001. High-risk rectal cancer is associated with a higher conversation rate of TSTC lesions to metastases than low-risk rectal cancers. Risk stratified; routine abbreviated liver MRI sequences should be investigated as part of the patient pathway for high-risk rectal cancer.

The incidence of colorectal cancer is increasing, with rectal cancer accounting for one-third of all cases.^1^ Increasingly personalized treatment options are available for rectal cancer, including the move toward a total neoadjuvant approach and the potential for watch and wait after complete response.^2–6^ Accurate staging for the presence of metastatic disease is crucial to ensure appropriate treatment from the outset, especially as increasingly, patients with small-volume metastatic disease can be treated with a curative approach.^7^

The liver is the commonest site of metastatic disease, and while there has been significant improvement in the treatment of the primary tumor, up to 50% of patients with colorectal cancer will develop metastatic disease in the liver and liver metastatic disease remains the commonest cause of death in this patient group.^7^

Staging for metastatic disease is routinely undertaken by contrast-enhanced computed tomography (CT). However, up to 40% of patients may require further characterization of the liver with magnetic resonance imaging (MRI) (on the basis of data from the VALUE trial)^8^ with Gd-EOB-DTPA-MRI liver imaging having the best sensitivity and specificity for the diagnosis of liver metastases.^8–10^ Other authors have explored the use of unenhanced liver MRI^11^ for the detection of liver metastases, including compared with contrast-enhanced ultrasound^12^ and the use of whole-body diffusion-weighted MRI as an alternative to CT staging.^13,14^

However, despite this evidence, routine MRI of the liver is not performed for most patients. Instead, many centers operate a pragmatic approach of performing MRI liver with either Doteram or hepatocyte-specific contrast (Gd-EOB-DTPA-MRI) and diffusion-weighted imaging (DWI) sequences for patients with indeterminate liver lesions on initial contrast-enhanced CT. This is supported by international guidance, for example, the ESMO practice guidelines recommend liver MRI to “characterize non-typical liver lesions on CT scans or when liver metastases seem resectable or potentially resectable.”^7^ While this approach allows for accurate characterization of those patients with too small to characterize (TSTC) lesions identified on CT, it leads to a delay in final staging, increased period of uncertainty for patients, and therefore a likely delay in the initiation of appropriate treatment. We report our experience undertaking routine diffusion weighted and T2 MRI sequences of the liver at the time of initial rectal MRI, and the potential for adopting a risk-stratified approach to the use of liver MRI. We looked to compare the incidence of synchronous liver metastases diagnosed by DW-MRI and ceMDCT in patients with MRI-defined high-risk versus low-risk rectal cancer.

## Patients and Methods

### Study Population

This retrospective study was approved by the Institutional review board of the Royal Marsden Hospital, London, UK. The requirement for informed consent from patients was waived because the research presented no risk of harm to the subjects, involved no procedures outside of their clinical care, and patients could not be identified individually.

Patients referred to an acute general hospital in the UK for the management of rectal cancer were imaged with high resolution rectal MRI and portal venous phase mulitdetector contrast enhanced CT (ceMDCT) as per the national guidelines.^15,16^ In addition, patients also underwent abbreviated non-contrast liver MRI to screen for liver metastases as per the hospital’s standard imaging protocol.

A total of 104 patients with rectal cancer were identified from a retrospective review of the records of patients discussed at multidisciplinary cancer management meetings. From April 2011 to May 2013, we selected 99 patients with rectal cancer who had undergone contrast-enhanced multidetector CT (ceMDCT), rectal MRI, and abbreviated liver MRI (as per standard of care in this hospital). The inclusion criteria for the study population were (1) patients with histopathologically proven adenocarcinoma of the rectum; (2) patients who had undergone ceMDCT of the thorax, abdomen, and pelvis; rectal MRI; and abbreviated liver MRI within 4 weeks; and (3) diagnostic quality imaging. Patient characteristics are described in Table 1. Due to the retrospective nature of data collection, reliable race and ethnicity data could not be provided.

**Table 1:** Scoring criteria for liver lesions on ceMDCT and abbreviated liver MRI

	Liver metastasis	Indeterminate/TSTC liver lesions	Normal/unchanged from previous/characteristic benign appearance
ceMDCT	Hypodense lesion with peripheral ring enhancement	Other lesion that was not seen on previous imaging but which did not meet the criteria for a liver metastasis.	No liver lesions or lesions unchanged from previous imaging or lesions with characteristic benign appearance.
Abbreviated liver MRI	Lesion with restricted diffusion (high signal) on the DWI sequence with a corresponding area of low signal on the ADC map. The signal intensity of the lesion on DWI should be higher than the signal intensity on the T2 weighted sequence	Liver lesion did not meet the criteria for with liver metastasis or normal/unchanged from previous	No liver lesions or lesions unchanged from previous imaging or those with characteristic benign appearance

### Imaging Protocols

#### CT Imaging Protocol

ceMDCT scans were performed on either a Phillips Brilliance 64 slice scanner (variable KV 100–140, smart mAs, auto collimation, reconstruction every 2.5 mm) or on a Phillips Ingenuity 128 slice scanner (variable kV 80–140, smart mAs, auto collimation, reconstruction every 2.5 mm) (Phillips, NV, USA). No oral contrast or water was given; 90 ml of intravenous iodinated contrast was administered at 3 ml per s. Images of the chest were obtained in the arterial phase and images of the abdomen and pelvis were obtained in the portal venous phase following the standard protocols for the staging of patients with rectal cancer at the hospital.

#### MRI Imaging Protocols

Rectal MRI scans were performed on a 1.5T Siemens Avanto scanner with a Synergy 6 channel phased array body coil (Siemens, UK) used in conjunction with a Synergy 6 channel phased array spine coil (Siemens, UK). Patients emptied their bladder and received 20 mg of intramuscular Buscopan before the examination. Patients were imaged in supine position. Sagittal T2, axial oblique, and coronal oblique sequences were performed with a slice thickness of 3 mm and 200 × 100 mm field of view were performed with a voxel size of 1.92 mm^3^ (0.8 × 0.8 × 3.0 mm). A large field of view (380 mm) axial T1 sequence of the pelvis with a slice sequence of 5 mm was also performed for characterizing incidental findings, with a voxel size of 9.6 mm^3^ (1.6 × 1.2 × 5.0 mm).

The abbreviated liver MRI scans were performed on a 1.5T Siemens Avanto scanner with a Synergy 6 channel phased array body coil (Siemens, UK) used in conjunction with a Synergy 6 channel phased array spine coil (Siemens, UK). T2 weighted haste axial T2 80 and EP 2D diffusion sequences at B50, B300, and B700 with an apparent diffusion coefficient (ADC) map were acquired. Patients were scanned in the supine position during free breathing, after the rectal MRI scan.

Rectal MRI and abbreviated liver MRI scans were performed as part of the standard rectal MRI protocol during one attendance.

### Image Analysis

ceMDCT was reviewed by one gastrointestinal radiologist (HH) with >10 years’ experience blinded to the original reports and the abbreviated liver MRI and rectal MRI but with access to patients prior imaging and clinical information as per normal practice. Abbreviated liver MRI and rectal MRI scans were reviewed in consensus by two specialist gastrointestinal radiologists with 7 and > 10 years’ experience (A.W., G.B.). Rectal MRI and abbreviated liver MRI were reviewed at least 14 days apart to reduce the chance for bias. Imaging was reviewed blinded to the ceMDCT imaging to address a further potential source of bias.

ceMDCT and abbreviated liver MRI were scored for the presence or absence of liver lesions. All liver lesions were scored as either liver metastases, indeterminate/TSTC lesions, or benign lesions according to the criteria outlined in Table 1. A final per-patient score was assigned as it would be in clinical practice, therefore, if the patient had both malignant and indeterminate/TSTC lesions on imaging, they would be scored as having had a malignant lesion.

A lesion was considered to be a true metastasis if one or more of the following criteria were met: (1) biopsy or resection of the lesion confirming metastasis, (2) progression of disease (enlargement of lesion by ≥ 20%), and (3) response to treatment (30% reduction in maximum diameter of the lesion following treatment).

**Fig. 1:**
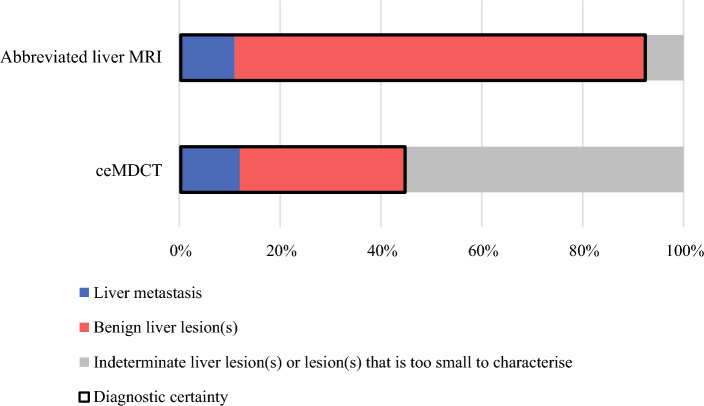
Spread and proportions of liver lesions by ceMDCT and abbreviated liver MRI; more liver lesions were demonstrated in total by abbreviated liver MRI (55 patients, compared with 42 patients who had a liver lesion demonstrated by ceMDCT); there was significantly greater diagnostic certainty by abbreviated liver MRI 93% (solid black box) compared with 45% by ceMDCT, *p* < 0.0001

**Fig. 2:**
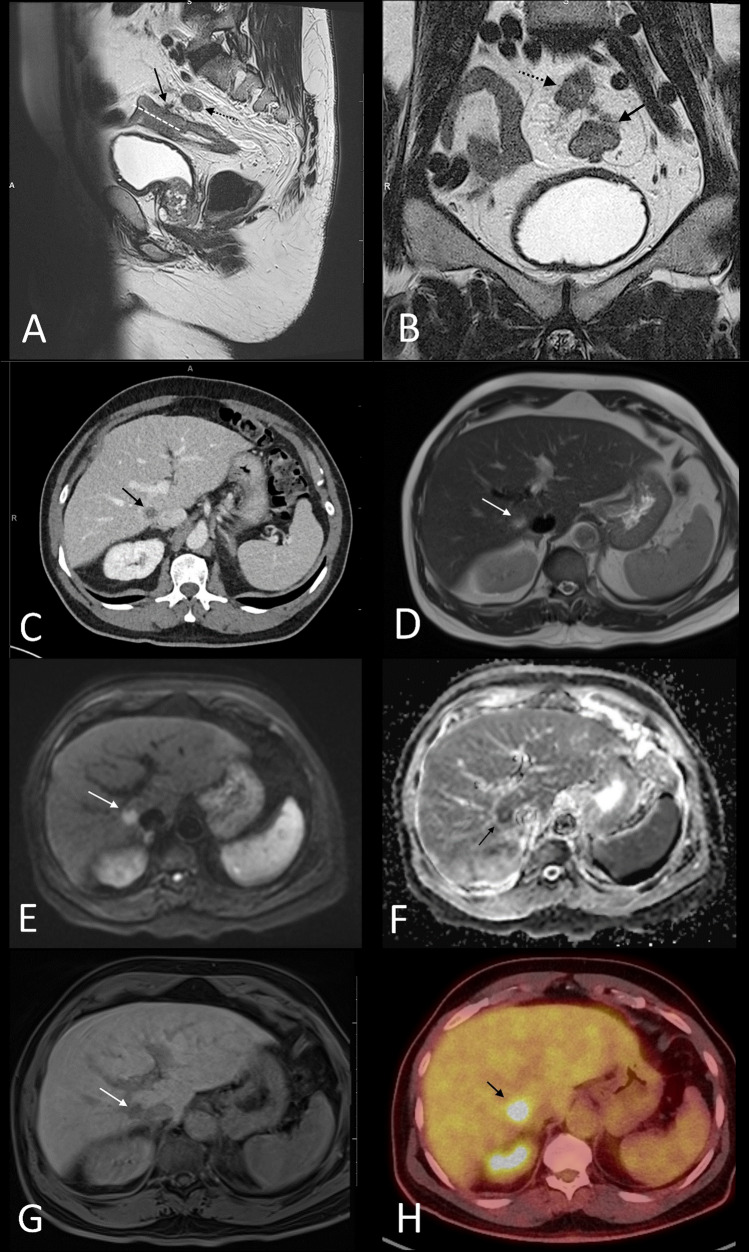
**A** and **B** sagittal and high-resolution coronal oblique images of a high-risk T3c N1c EMVI-positive CRM-positive PSW-negative upper rectal tumor; dashed white line delineates the maximal length of the tumor, solid black arrow the EMVI, and dashed black arrow the vascular deposit (N1c); **C** contrast-enhanced CT showing a hypodense lesion in the right lobe of the liver but without definite peripheral enhancement to confirm the presence of liver metastasis; **D** T2-weighted MRI of the liver showing the lesion to be high signal; (**E**) and (**F**) B800 series of the diffusion weighted sequence, **E** showing the liver lesion to be high signal with corresponding low signal on the ADC map and **F** confirming the lesion restricts diffusion; **G** 25-min (delayed) hepatocyte specific contrast-enhanced MRI showing wash-out confirming the lesion to be a metastasis, no further metastases are demonstrated; **H** PET-CT showing the lesion is FDG avid again, confirming the lesion to be a metastasis; in this case abbreviated liver MRI confirmed the lesion identified on CT to be suspicious for a metastasis and that there were no other suspicious liver lesions

Patients were stratified into high- and low-risk groups according to findings of the rectal MRI according to the previously published reporting criteria^17^ (Figs. 1 and 2). Patients were stratified as high risk if any of the following validated poor prognostic features was present: (1) ≥ 5 mm extramural spread, (2) medium or large vessel extramural venous invasion, and (3) involvement of the circumferential resection margin (< 1 mm) or involvement of the intersphincteric plane for low rectal tumors.

### Statistical Analysis

The primary outcome was to compare the prevalence of synchronous liver metastases diagnosed by abbreviated liver MRI in patients with MRI-defined high-risk versus low-risk rectal cancer. The effect of potential confounders and effect modifiers were not modeled as the imaging was reviewed at diagnosis before any impact of these effects, and the aim of the study was to assess the effect of stratification by MRI at baseline. On the basis of previous literature^18^ with an expected prevalence of 4% in the low-risk group, showing a rate of at least 12% in the high-risk group would be considered significant with a sample size of 99 patients. Differences between groups were assessed using the chi-squared test or one-sided Fisher’s exact test as appropriate. No continuous variables were assessed.

Descriptive statistics were used to describe diagnostic certainty, defined as confidently diagnosed liver metastases or benign lesions on imaging.

A total of 10 patients were lost to follow-up within 12 months, but this did not affect the primary outcome, which looked at baseline imaging. The baseline characteristics of the whole cohort, 89 patients with 1 year of follow-up data and 10 patients lost to follow-up, are given in Table 2; 8 patients had no further treatment at this center (presumed care transferred to a more local center), 1 had best supportive care only, and 1 refused treatment.

**Table 2:** Patient demographics

Total (*n*)		Whole cohort		Patients with 1-year follow-up		Patients lost to follow-up within 1 year of diagnosis	
		99		89		10	
Characteristic		*n*	%	*n*	%	*n*	%
Gender	Female	33	33%	28	31%	5	50%
	Male	66	66%	61	69%	5	50%
Age at diagnosis	< 60 years	15	15%	15	17%	0	0%
	≥ 60 years	84	85%	74	83%	10	100%
Height of primary tumor (on MRI)	Low rectal (< 5 cm)	25	25%	22	25%	3	30%
	Mid rectal (5–10 cm)	51	52%	45	51%	6	60%
	Upper rectal (10–15 cm)	23	23%	22	25%	1	10%
mrT stage	T1	25	25%	21	24%	4	40%
	T2	18	18%	17	19%	1	10%
	T3a	6	6%	5	6%	1	10%
	T3b	15	15%	14	16%	1	10%
	T3c	14	14%	13	15%	1	10%
	T3d	9	9%	8	9%	1	10%
	T4	12	12%	11	12%	1	10%
mrN stage	N0	51	52%	45	51%	6	60%
	N1	9	9%	9	10%	0	0%
	N2	3	3%	3	3%	0	0%
	N1c	36	36%	32	36%	4	40%
mrEMVI status	Negative	54	55%	48	54%	6	60%
	Positive	45	45%	41	46%	4	40%
mrCRM status	Negative	84	85%	75	84%	9	90%
	Positive	15	15%	14	16%	1	10%
mrISP plane (low rectal tumors only, *n* = 25	Negative	15	60%	13	15%	3	30%
	Positive	10	40%	9	10%	1	10%
Summary risk (from rectal MRI staging)	High risk primary	46	46%	42	47%	4	40%
	Low risk primary	53	54%	47	53%	6	60%

Survival estimates for overall survival were obtained using the Kaplan–Meier product limit method. Patients were censored at their last follow-up. Statistical analysis was performed by SPSS 25.0.0 (SPSS, Chicago, IL) and Medcalc Software 2019. The STROBE Statement was completed for this study (in the Supplementary Material).

## Results

A total of 99 eligible patients were included; 66 were male (67%). Mean age was 70 years (range 38–89 years). All patients had ceMDCT, abbreviated liver MRI, and rectal MRI at diagnosis, and 46/99 (46%) had a high-risk primary rectal cancer, 42/99 (42%) had a liver lesion seen on ceMDCT, and 55 (56%) had a liver lesion seen on abbreviated liver MRI.

### Liver Lesions in All Patients

On a per-patient level (where patients were categorized by their most significant liver lesion) CT demonstrated 5 patients with liver metastases, 23 patients with indeterminate or TSTC liver lesions, and 14 patients with benign liver lesion(s) only. The five patients with liver metastases demonstrated on ceMDCT had these confirmed by abbreviated liver MRI. Of the 23 patients with indeterminate/TSTC liver lesions by ceMDCT, 1 was diagnosed as a liver metastasis by abbreviated liver MRI and the remainder were characterized as benign by abbreviated liver MRI.

Abbreviated liver MRI demonstrated 6 liver metastases (the 5 patients with liver metastases diagnosed by ceMDCT and 1 patient with an indeterminate/TSTC lesion on ceMDCT), 45 patients with benign liver lesions, and 4 patients with indeterminate/TSTC lesions.

A total of 10 patients were lost to follow-up, leaving 89 patients (42 high-risk primary rectal cancer, 47 low-risk) with 12-month follow-up, this included all those with malignant or liver lesions on abbreviated liver MRI. Of the four indeterminate lesions on abbreviated liver MRI, three were confirmed as benign on follow-up and one as malignant (outcomes are described in Table 3). By 12 months, a further sixpatients had developed liver metastases (all of whom had high-risk rectal cancer at baseline).

**Table 3:** The 12-month outcomes of all the malignant and indeterminate/TSTC liver lesions demonstrated by abbreviated liver MRI at baseline at 12 months; no missing patients

High- or low-risk primary rectal tumor	Malignant or indeterminate/TSTC lesions on abbreviated liver MRI	ceMDCT appearance of liver lesion	Outcome at 12 months
High	Malignant	Malignant	Malignant
High	Malignant	Malignant	Malignant
High	Malignant	Malignant	Malignant
High	Malignant	Malignant	Malignant
High	Malignant	Indeterminate	Malignant
High	Malignant	Malignant	Malignant
Low	Indeterminate	No lesion	Malignant
Low	Indeterminate	No lesion	Liver lesion found to be a vascular perfusion defect on contrast-enhanced MRI. Not identified on follow-up CT or MRI.
High	Indeterminate	No lesion	Benign liver lesion—unchanged on subsequent follow-up
High	Indeterminate	Benign	Liver lesion had benign characteristics on CT but was indeterminate on DW-MRI

#### Diagnostic Certainty

Abbreviated liver MRI provided diagnostic certainty (defined as confidently diagnosed liver metastases or benign lesions on imaging at baseline) in 51/55 patients (93%) compared with in only 19/42 patients (45%) by ceMDCT. Thus, there was a 48% (95% CI 30–63%, *p* < 0.0001) difference in diagnostic certainty when the liver was staged with abbreviated liver MRI, Fig. 1.

### Risk of Liver Lesions according to Risk of the Primary Tumor

**Fig. 3:**
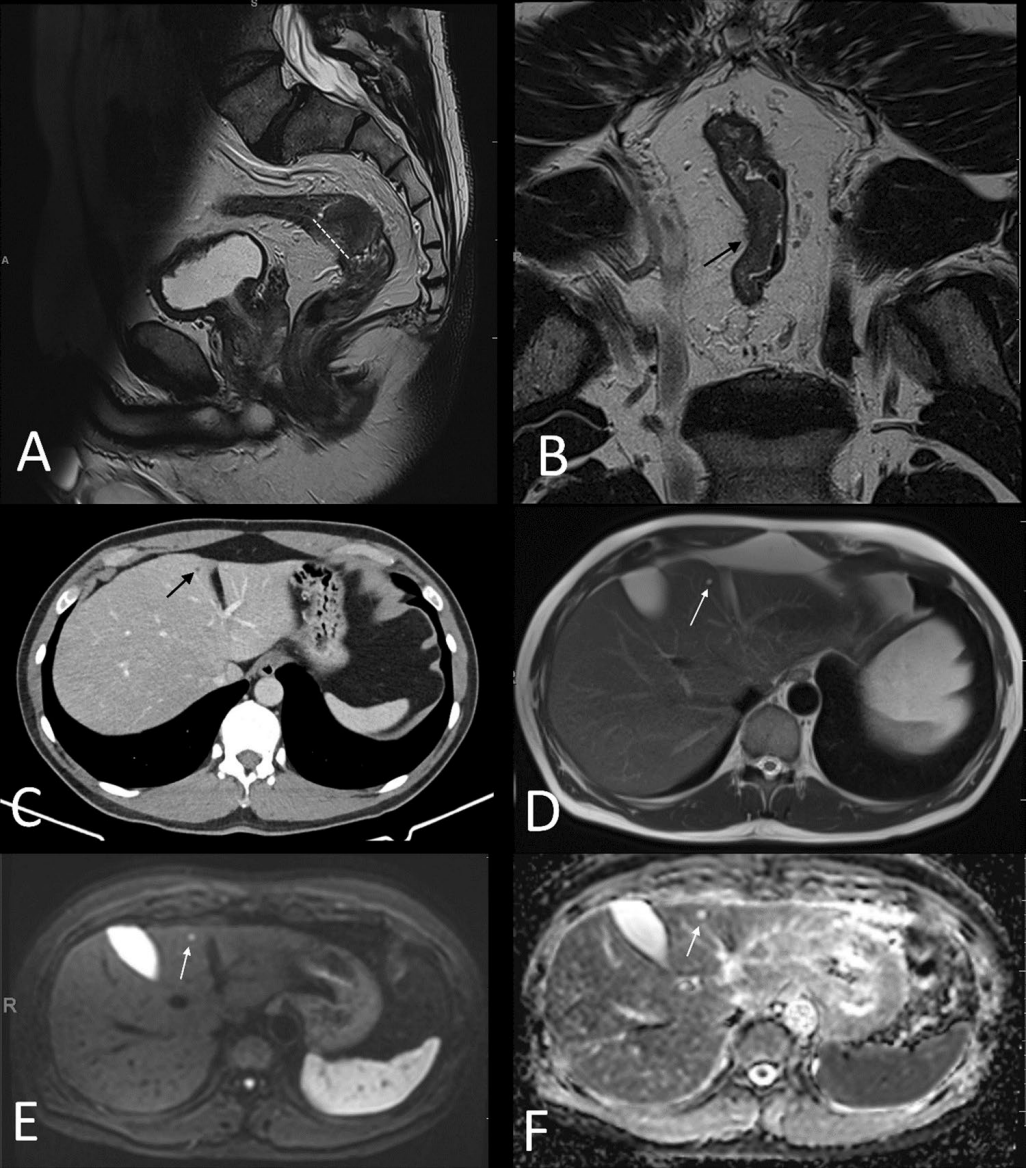
**A** and **B** sagittal and high-resolution coronal oblique images of a low-risk T3b N0 EMVI-negative CRM-negative PSW-negative mid-rectal tumor; dashed white line delineated the maximal length of the tumor, solid black arrow shows the 3 mm of extramural spread consistent with T3b disease; **C** contrast-enhanced CT that showed multiple too small to characterize hypodense liver lesions; **D** T2-weighted MRI of the liver that showed multiple small high T2 liver lesions, more numerous than shown on the CT; **E** and **F** B400 series of the diffusion weighted sequence (**E**) and the corresponding ADC map (**F**) showing the multiple liver lesions to be high signal on both sequences consistent with T2 shine through and not true restricted diffusion, confirming the lesions to be small, benign hepatic cysts; there were no suspicious lesions identified; in this case abbreviated liver MRI confirmed the multiple too small to characterize lesions identified on CT to be benign

In total, 46 (46%) of the patients had a high-risk primary rectal cancer and 42 (54%) had a low-risk primary rectal cancer. High-risk rectal cancers had a trend to higher risk of liver metastases confirmed at baseline on both ceMDCT (OR 14.18) and on abbreviated liver MRI (OR 17.18), but this did not reach statistical significance (*p* = 0.08 and 0.06, respectively, Table 4). There was no difference in the total number of lesions, indeterminate/too small to characterize, or benign lesion(s) diagnosed by ceMDCT or abbreviated liver MRI. Examples are provided in Figs. 2 and 3.

**Table 4:** Liver lesions on ceMDCT and abbreviated liver MRI according to the risk of the primary rectal cancer

	ceMDCT					Abbreviated liver MRI				
	All patients	High risk primary	Low risk primary	OR (95% CI)	*p*-value	All patients	High risk primary	Low risk primary	OR (95% CI)	*p*-value
	*n* = 99	*n* = 46	*n* = 53			*n* = 99	*n* = 46	*n* = 53		
	*n* (%)	*n* (%)	*n* (%)			*n* (%)	*n* (%)	*n* (%)		
All liver lesions	42 (42)	19 (41)	23 (43)	0.91 (95% CI 0.41–2.04	0.83	55	26	29	1.08 (95% CI 0.49–2.38)	0.86
Liver metastasis	5 (5)	5 (11)	0 (0)	14.18 (95% CI 0.76–263.81)	0.08	6	6	0	17.18 (95% CI 0.94–313.77)	0.06
Indeterminate/TSTC	23 (23)	8 (17)	15 (28)	0.53 (95% CI 0.2–1.41)	0.2	4	2	2	2.43 (95% CI 0.42–13.92)	0.32
Benign lesions only	14 (14)	6 (13)	8 (15)	0.84 (95% CI 0.27–2.64)	0.7	45	18	27	0.62 (95% CI 0.28–1.38)	0.24

## Discussion

We aimed to describe our experience of the routine use of abbreviated liver MRI for the diagnosis of synchronous liver metastatic disease at baseline in patients with rectal cancer and whether the risk of the primary tumor determined the risk of liver metastases diagnosed by abbreviated liver MRI.

Abbreviated liver MRI diagnosed more liver metastases than ceMDCT and fewer indeterminate/TSTC lesions, and thus provided diagnostic certainty in 93% of patients with liver lesions compared with only 45% by ceMDCT (*p* < 0.0001). All six liver metastases diagnosed at baseline by either ceMDCT or abbreviated liver MRI were in patients with high-risk rectal cancer consistent with prior literature,^18^ although this did not reach statistical significance in our cohort, likely secondary to sample size. Previous work has shown that MRI staging of the primary rectal cancer on the basis of criteria other than tumor, node, metastasis (TNM), such as extramural venous invasion (EMVI) positivity,^19–21^ circumferential resection margin (CRM) involvement,^22–24^ and involvement of the low rectal cancer plane,^25^ successfully identifies patients at risk of recurrence. Our work adds evidence to the value of rectal MRI in predicting patients who subsequently develop distant metastatic disease and therefore who would benefit from preoperative treatment as well as more intensive follow-up, such as abbreviated liver MRI.

Other authors investigating the impact of routine liver MRI for all patients with newly diagnosed colorectal cancer found the diagnostic yield of liver metastases was very low (2.2%) in those with a normal CT or a CT that showed liver lesions that were too small to characterize (TSTC).^26^ However, patients with TSTC liver lesions represent a significant diagnostic dilemma to tumor boards, with 13–30.2%^26–28^ of patients with colorectal cancers having these lesions on initial CT, although the literature to date suggests these lesions are normally benign.^27,29^ However, the early diagnosis of small volume synchronous liver metastatic disease may indicate poor prognostic tumor biology,^30^ changes the patient treatment pathway (providing options for early access to systemic chemotherapy), and thus improves outcomes, enabling liver resection.^31^ Therefore, radiologists and tumor boards remain nervous dismissing these lesions without definitive imaging. In our cohort, 23 indeterminate or TSTC lesions were demonstrated by ceMDCT, one of which was confirmed as a liver metastasis by abbreviated liver MRI at diagnosis, and a further indeterminate lesion had benign characteristic on MRI at baseline but then progressed on follow-up and was determined to be a metastasis. At baseline there was therefore a 4% conversion rate of TSTC lesions on ceMDCT.

However, when the cohort of ceMDCT is divided by those with high- and low-risk rectal cancer, the conversion rate at diagnosis is 12.5% (1/8) in high-risk rectal cancers compared with 0% (0/15) in low-risk cancers. To the best of our knowledge, these are the first data to show this. A risk-stratified approach to offering liver MRI may therefore prove a cost-effective model for characterization of liver lesions in this at-risk cohort with a higher rate of liver metastases detected and increased reassurance for indeterminate liver lesions. The consideration of risk-stratified approaches to imaging is timely given the increasing pressures on imaging services.

Staging for liver metastases is routinely undertaken by CT, however, the sensitivity of CT for liver metastases is ~70%.^32^ There have been multiple meta-analyses reporting the sensitivity and specificity of contrast-enhanced CT,^33^ abbreviated liver MRI based on DW-MRI sequences, and hepatocyte-specific contrast-enhanced MRI,^34^ including Wu et al., who reported contrast enhanced MRI and DWI as having equivalent sensitivities for the diagnosis of liver metastases, 0.9 versus 0.87 (*p* > 0.05), but a combination of DWI-MR and contrast-enhanced MRI combined has the best sensitivity for the diagnosis of liver metastases with a sensitivity of 97%.^34^ Zech et al. reported that 39.7% of patients staged with contrast-enhanced CT required further imaging of the liver within the VALUE trial (a cohort of patients with confirmed or suspected liver metastases)^9^ and reported that the cost of diagnostic workup was lower when a hepatocyte-specific MRI was used as the initial imaging procedure for the detection of colorectal cancer liver metastases.^8^ However, within the UK, the use of routine MRI for the detection of liver metastases is unlikely to be adopted, as fewer MRI scanners and radiologists are available.

Performing abbreviated, non-contrast liver MRI at the initial staging scan could be a solution. In our protocol, the non-contrast MRI sequences rely on the use of DW-MRI sequences for the detection of liver metastases, with anatomic T2 weighted sequences used for anatomical localization. Multiple authors, systematic reviews, and meta-analyses have reported the improved sensitivity of whole-body DWI for detection of liver metastases^12,35^ and others have explored the possibility of replacing contrast-enhanced CT with MRI.^13^ Other authors have shown comparable sensitivity of abbreviated liver MRI protocols (on the basis of DW-MRI) compared with the gold standard examination of hepatocyte-specific contrast-enhanced MRI of the liver,^10^ and some have explored abbreviated liver MRI in follow-up,^36^ but again with contrast-enhanced sequences. Similarly, routine MRI for the detection of liver metastases has also been reviewed for pancreatic cancer,^37^ but despite this, contrast-enhanced CT remains the standard method of staging, which is likely in no small part secondary to the ease of the established patient pathways on the basis of CT staging and the improved staging of liver metastases, but also the challenges of scheduling additional MRI scans in already stretched services. Other authors have looked at the use of abbreviated liver MRI protocols for the diagnosis and restaging of colorectal liver metastatic disease.^36^ The challenge now is to introduce abbreviated liver MRI pathways to the patient workflow, and to those who are most likely to benefit. The relative cost of staging pathways has been reviewed outside the UK,^8^ but not within the UK.

Performing abbreviated liver MRI sequences at the initial staging scan can prevent patients from having a gadolinium-enhanced scan unnecessarily, provides immediate reassurance regarding TSTC liver lesions seen on ceMDCT, and identifies those patients who need to have a contrast-enhanced liver MRI to assess metastatic load and guide further treatment options. The identification of high-risk rectal cancers using the MR stratification (who are at risk of liver metastases) could be used in conjunction with circulating DNA of minimal residual disease to improve the timing of abbreviated liver MRI studies and earlier detection of hepatic metastatic disease.

This study is limited by describing single-center experience but by necessity as abbreviated liver MRI at diagnosis is not routine in the UK. The sample size was predetermined according to the available literature. This cohort contains 25% of patients with T1 disease, which may not reflect the patient population seen currently in all centers, but with increasing adoption of bowel cancer screening earlier stage tumors are being identified,^38^ and therefore this cohort is very relevant.

In total, ten patients (10%) were lost to follow-up within 12 months of diagnosis, for the majority because care was presumed to have been moved to another center (due to the nature of regionalized bowel cancer screening within the UK, patients can choose to have their treatments at another center), however, this does not impact upon the primary endpoint, which looked at baseline staging and diagnostic certainty. The spread of patients lost to follow-up matches that of the whole cohort and those with 12 months of follow-up and therefore does not introduce additional bias.

As this study describes single-center experience of using abbreviated MRI in a clinical setting, patients without a malignant or indeterminate liver lesion(s) on abbreviated MRI did not receive further liver imaging at baseline, and thus, without contemporaneous gold standard liver imaging in all patients at baseline, meaningful sensitivity and specificity calculations for abbreviated MRI and contrast-enhanced CT could not be made. There is no reason to assume sensitivity and specificity would be different in our study to the published meta-analyses (described above), but it could not be reliably formally assessed.

We chose to report lesions on a per-patient rather than per-lesion basis. This was a conscious decision, as the presence of one metastatic or indeterminate lesion would change the patient pathway and thus our data represent the dilemma faced by tumor boards weekly. Detailed scoring of the imaging appearances and size of the liver lesions was not made beyond their classification as malignant, indeterminate/TSTC, or benign by abbreviated liver MRI or contrast-enhanced CT, as the aim of this study was to determine the added clinical benefit of abbreviated liver MRI, but this would be a useful piece of work for the future. Similarly, interobserver variability was not assessed in this study but has been shown to be excellent in other studies.^39,40^

Finally, we considered only the staging of the liver at baseline. Further work is needed to determine how risk stratification of the primary tumor determines the risk of metachronous liver metastases and if and when abbreviated liver MRI surveillance should be considered.

## Conclusions

Abbreviated liver MRI diagnosed fewer indeterminate/too small to characterize lesions than ceMDCT and thus provided greater diagnostic certainty than scanning with ceMDCT. High-risk rectal cancers are associated with a higher conversation rate of TSTC lesions to liver metastases than low-risk rectal cancers. Risk-stratified abbreviated liver MRI sequences should be investigated as part of the patient pathway for high-risk rectal cancer. Thus, patients are not exposed to gadolinium unless required, radiology stocks of contrast are maintained, and costs are lowered, while providing patients with improved care.
